# Low practice of malaria prevention among migrants and seasonal farmworkers in Metema and west Armacheho districts, Northwest Ethiopia

**DOI:** 10.1186/s12879-021-05853-x

**Published:** 2021-02-04

**Authors:** Getu Debalkie Demissie, Tadesse Awoke Ayele, Sintayehu Daba Wami, Malede Mequanent Sisay, Destaw Fetene, Haileab Fekadu Wolde, Temesgen Yihunie Akalu, Kassahun Alemu Gelaye

**Affiliations:** 1 Department of Health Education and Behavioral Science, Institute of Public Health, University of Gondar, Gondar, Ethiopia; 2 Department of Epidemiology and Biostatistics, Institute of Public Health, University of Gondar, Gondar, Ethiopia; 3 Department of Environmental and Occupational Health and Safety, Institute of Public Health, University of Gondar, Gondar, Ethiopia

**Keywords:** Knowledge, Practice, Attitude, Malaria, Migrant and seasonal farm workers

## Abstract

**Background:**

More than hundreds and thousands of migrants and seasonal farm workers move from the highlands (relatively low malaria endemicity areas) to the lowlands (higher malaria endemicity areas) for the development of the corridor of the Amhara region during planting, weeding, and harvesting seasons in each year. Seasonal migrant workers are at high risk of malaria infection. Therefore, evidence of their knowledge level and practice in the prevention of malaria during their stay would be important.

**Objective:**

The aims of this study was to assess the knowledge and practice of malaria prevention and associated factors among migrants and seasonal farm workers in Northwest Ethiopia.

**Method:**

A cross-sectional study was conducted from October to November, 2018 in Metema and West Armacheho districts, northwest Ethiopia. A sample of about 950 migrants and seasonal farm workers were included using two stages of cluster sampling technique. Interview administered structured questionnaire was used. Both bi-variable and multivariable binary logistic regressions were applied to identify predictors of malaria prevention.

**Result:**

The overall good knowledge of malaria (those participants who scored more than 60% of correct response for knowledge related questions) was 50.2% with 95% CI (47.0–53.0) and the overall good practice of malaria (those participants who practiced more than 60% for practice related questions) was 27.2% with 95% CI (244.3–29.9). Age (AOR = 0.51(95%CI; 0.33–0.80)), level of education (AOR = 0.55(95%CI; 0.32–0.94)), using mass media as a source of information (AOR = 2.25(95%CI; 1.52–3.32)) and length of stay at the farming site (AOR = 0.59(95%CI; 0.44–0.79)) were significantly associated with knowledge of malaria prevention. Knowledge (AOR = 6.62(95%CI; 4.46–9.83)), attitude (AOR = 2.17(95%CI1.40–3.37), use of mass media (AOR = 1.64(95%CI; 1.30–2.60)) and the length of stay (AOR = 1.93(95%CI; 1.35—2.77)) in the farming area were significantly associated with practice of malaria prevention.

**Conclusion:**

The practice of malaria prevention among migrant and seasonal farm workers was low. The programmers and implementers should design tailored malaria intervention programs and strategies for these hard to reach population.

## Introduction

World Health Organization (WHO) reported that 3.2 billion people remained at risk of malaria in 2016. In the same report, globally, there were 212 million malaria cases and 4 million deaths, of which 92% were in African region [1]. Malaria is placed a leading communicable disease in Ethiopia, accounting for about 30% of the overall DALY (Disability Adjusted Life Years) lost [2].

Recent studies have shown that history of travel is a risk factor for malaria in some parts of Ethiopia [3, 4]. Migrant and seasonal farmworkers (MSFWs) are one of the high risk groups for malaria [5]. The Government of Ethiopia recommended to take weekly mefloquine administered at 5 mg/kg drug for chemoprophylaxis for non-immune travelers who visit malarias areas for a period of 2–3 month. Chemoprophylaxis should be started 2 weeks before departure and 4 weeks after return from the malaria risk areas [6].

In each year**,** an estimated 400,000–500,000 MSFWs move from the highlands (relatively low malaria endemicity areas) to the lowlands (higher malaria endemicity areas) of Amhara region (Ethiopia) during planting, weeding, and harvesting seasons. Metema and West Armacheho (the largest scale agricultural farming area in the region) accounted for 20.9% of all confirmed malaria cases from the Amhara region, and the prevalence of malaria in this region is 12% [7].

MSFWs perform their daily activities based on a contract basis. As a result, they work for a long hours including nightfall. This working conditions expose them for malaria mosquito’s bite. They are also at risk of bringing malaria infection back to their home communities [8]. It was discovered that untreated malaria could result into hepatomegaly, splenomegaly, varying degree of anemia and various syndromes resulting from the involvement of individual organs [9].

Although the knowledge and practice of malaria prevention play a significant role to prevent malaria, there are limited studies which explains the knowledge and practice of malaria prevention among migrant and seasonal farm workers.. Therefore, the aim of this study was to assess the knowledge and practice of malaria prevention and associated factors among MSFWs in Metema and West Armacheho districts, northwest Ethiopia which in turn provides evidences for the programmers and implementers to consider these population in designing malaria elimination intervention strategies.

## Methods

### Study design and setting

A cross-sectional study was conducted from October to November 2018. The study was conducted in Metema and West Armacheho districts, northwest Ethiopia which are some of the fertile agricultural areas with a large scale of the farming of cash crops in the Amhara region. As a result, hundreds and thousands of migrants and seasonal farm workers travelled to these areas to work, especially in the weeding and harvesting seasons. The area is endemic with malaria, and seasonal farm workers are most affected [10].

### Study participants, sample size and sampling procedure

The source population were all migrant and seasonal farm workers who move to these farming areas for temporary farming work. A sample of about 950 migrants and seasonal farm workers participated in the study using two stages of clustered sampling technique. Pilot study was conducted among 50 migrants and seasonal farm workers in Quara district, West Gondar zone to determine the minimum sample size.

The sample size was determined using single population proportion formula by using the following assumptions. According to the pilot study, the proportion of knowledge and practice were 44, and 34%, respectively. The assumption were 95% level of confidence, 5% margin of error, design effect of 2 and 5% non-response rate.

Since the sampling technique was cluster, design effect of 2 was used so that the total sample size of 804, and 702 was calculated for knowledge and practice respectively. The sample size was also computed for predictors of health information (OR = 1.7), occupation (OR = 1.5) and favourable attitude (OR = 1.92) with the assumptions of power, 80 and 95% CI and the sample size were 976, 870 and 648 respectively. Finally, the largest sample size of 976 was used.

There were 174 farm sites in the two districts. The average number of migrant and seasonal laborers in each farm is estimated to be 460. In the first stage of sampling, primary sample units (PSUs) were formed by segmenting the farm sites so that each PSU would include approximately 460 migrant and seasonal farm workers. A total of 174 PSUs was formed; of these, 14 farms/PSUs were referred to as “study sites”, and they were selected by lottery method. The required study participants from each study site was determined by systematic random sampling.

### Data collection and procedures

Piloted and Interview administered structured questionnaire was used. The questionnaire was developed from different literatures and administered through face to face interview. The interview was conducted by 10 BSc degree trained data collectors. The questionnaire included socio demographic characteristics of the participants, knowledge, practice and treatment seeking behavior related questions to malaria which were developed from different literatures. The questionnaire employed 30 items for knowledge with “yes” and “no “which were used to elicit information on knowledge of malaria transmission, preventive strategies and sign and symptom of malaria disease. The questionnaire also included 5 items for the practice of the preventive strategies with “yes” and “no “and 5 items for attitude with likert scale.

The instrument was drafted in English, and it was translated to Amharic. It was also translated back to English by language experts. Besides, a pilot study was conducted. A 2 day training was given to all data collectors and supervisors before the data collection.

### Operational definition of terms

Migrant and seasonal farmworker was defined as individuals who are required to be absent from a permanent place of residence for the purpose of seeking employment in agricultural work. “Good Knowledge” was defined as those participants who scored more than 60% of correct response for knowledge related questions. “Good practice” was defined as those participants who practiced more than 60% for practice related questions. “Favorable attitude” was defined as those participants who had positive attitude towards 60% of attitude related questions [11].

### Data analysis

Epi- data version 3.1 was used to enter the data and STATA version 12 was used for data analysis. Descriptive statistics including frequency distributions and tables were used to present the characteristics of the data. Bi-variable and multivariable binary logistic regressions were applied to determine the association between variables and the two different outcomes (good knowledge and good practice) in each logistic regression model. Variables which were found to have an association at *p*-value ≤0.2 with the dependent variable were entered into multivariable logistic regression for controlling the possible confounding effect. Finally, the variables were taken as significant by considering 5% level of significance. Adjusted odds ratio (AOR) with its respective 95% CI was reported to measure strength of association.

## Results

### Socio-demographic characteristics of participants

Out of the 976 migrant and seasonal farm workers, 950 of them completed the questionnaire with response rate of 97.3%. The mean age of the participants was 26 (SD: 7.82) years, in which most of them (96.6%) were from Amhara region. About 406 (42.7%) of the participants were unable to read and write and as 672(70.7%) of them were farmers. Nearly one in three (30.8%) of the participants visited the farming area 2–4 times (Table 1).

**Table 1:** Socio-demographic characteristics of seasonal migrant farmworkers in Metema and West Armacheho districts, Northwest Ethiopia, 2018(*n* = 950)

Variable	Frequency	Percentage
**Region**		
Amhara	918	96.6
Tigray	27	2.9
Oromia	5	0.5
**Sex**		
Male	943	99.3
Female	7	0.7
**Age**		
13–20	244	25.7
21–29	485	51.0
30–39	151	15.9
40–67	70	7.4
**Level of education**		
Unable to read and write	374	39.4
able to read and write	73	7.7
primary	406	42.7
secondary and above	97	10.2
**Occupation**		
Farmers	672	70.7
student	177	18.6
unemployed	101	10.7
**Family size**		
1–2	88	9.3
3–5	536	56.0
6–14	326	34.7
**Residence**		
Rural	832	87.6
Urban	118	12.4
**Religion**		
Orthodox	932	98.1
Others	18	18.9
**Marital status**		
Married	301	31.7
Non-married	649	68.3
**Income (daily as MSFWs)**		
Low	495	52.1
High	455	47.9
**No of visit**		
1 time	178	18.7
2–4	293	30.8
5–8	281	29.6
9–30	198	20.9
**Length of stay**		
Less than 2 months	576	60.3
More than 2 months	374	39.7

### Source of Health information

From the participants, 59.6% heard information related to malaria. Of these, 53.8% got the information from health workers, 34.5% from mass media and 28.4% from their friends.

### Knowledge of migrant and seasonal farmworkers about malaria

The overall good knowledge about malaria was 50.2%. From the participants, 79.2 and 73.8% of them mentioned mosquito bite as mode of transmission and risk factor for malaria respectively. The majority of the study participants mentioned sleep under ITN (Insecticide treated nets) as malaria prevention method followed by fill stagnant water**.** Fever, chills and rigor were the most frequently mentioned sign and symptom of malaria by the study participants (Table 2).

**Table 2:** Knowledge of participants about malaria in Metema and West Armacheho, Northwest Ethiopia, 2018 (*n* = 950)

Mode of transmission	Frequency (yes)	Percentage
Mosquito bite	752	79.2
Blood transfusion	114	12
Mother to child	62	6.5
**Risk factors**		
Dirty and stagnant water	701	73.8
Seasonal change	657	69.2
Infected mosquito bite	527	55.7
**Prevention methods**		
Sleep under ITN	728	76.6
Use mosquito repellant	385	40.5
Avoid mosquito bites	390	41
Take prophylaxis	200	21
Spray house with insecticide	428	45
Cut the grass around the house	541	57
Fill in puddles (stagnant water)	557	58.6
Keep house and its surroundings clean (clay, pot, leaves, …)	507	53.4
Put mosquito screens on the windows	324	34
**Sign and symptom**		
Fever	821	86
Headache	793	83.5
Nausea	531	55.9
Vomiting	557	58.6
Chills and rigor	840	88.4
Loss of appetite	689	72.5
Body ache or joint pain	734	77.3
Excessive sweeting	529	55.7
Body weakness	639	77.3

### Practice of malaria prevention and treatment seeking behavior

Of the participants, 258(27.2%) of them had good practice of different malaria prevention strategies. From these, only 11% of them used chemoprophylaxis and 20.7% of them utilized ITN in the last night. Only 214(22.5%) have ITN and from this, only 14% of the farmworkers received their ITN from a government mass distribution. The major reason for not utilizing ITNs was lack of accessibility (41%). Of the participants, 824 (86.7%) of them have ever had malaria in the working area. From these participants who ever had malaria, 649(78.8%) had no treatment seeking behavior and 559(67.8%) of them had gone to get health services in serious stage of the diseases.

### Factors associated with good Knowledge about malaria

The overall knowledge about malaria prevention was 50.2% with 95% CI (47–53%). The odds of having good knowledge is decreased by 41% among participants who lived less than 2 months in the farming area as compared with participants who lived more than 2 months. The odds of having good knowledge is increased by 2.25 times among participants who had media exposure as source of information for malaria than their counter parts (Table 3).

**Table 3:** Factors associated with good knowledge about malaria among seasonal and migrant farmworkers in Metema and West Armacheho districts, Northwest Ethiopia, 2018

Variables	Knowledge status		COR with 95%CI	AOR with 95%CI
	Good	Poor		
**Age**				
21–29	250	235	1	1
13–20	103	141	0.69 (0.50–0.94)	0.66 (0.46–0.95)*
30–39	91	60	1.43 (0.98–2.06)	1.28 (0.82–2.00)
40–67	33	37	0.84 (0.50–1.38)	0.88 (0.49–1.58)
**Level of education**				
Unable to read and write	164	210	0.44 (0.28–0.70)	0.55 (0.32–0.94)*
Able to read and write	44	29	0.86 (0.46–1.60)	0.90 (0.44–1.85)
Primary	207	199	0.59 (0.37–0.93)	0.70 (0.42–1.16)
Secondary and above	62	35	1	1
**Family size (in number)**				
1–2	49	39	1	1
3–5	280	256	0.87 (0.54–0.37)	1.10 (0.66–1.83)
6–14	148	178	0.66 (0.41–1.06)	0.89 (0.52–1.51)
**Marital status**				
Non-married	307	342	1	1
Married	170	131	1.44 (1.09–1.90)	1.34 (0.94–1.93)
**Salary (Daily as MSFW)**				
Low	264	231	1.30 (1.00–1.68)	1.29 (0.97–1.71)
High	213	242	1	1
**Number of times have you been her**				
1 times	95	83	1.27 (0.84–1.90)	1.35 (0.84–2.16)
2–4 times	146	147	1.10 (0.77–1.58)	1.19 (0.78–1.80)
5–8 times	142	139	1.13 (0.79–1.63)	1.20 (0.51–1.51)
9–30 times	94	104	1	1
**Length of time living here in the current visit**				
< 2 months	275	301	0.78 (0.60–1.00)	0.59 (0.44–0.79)*
> 2 months	202	172	1	1
**Using mass media (as source of information about malaria)**				
Yes	231	97	3.64 (2.73–4.84)	2.25 (1.52–3.32)*
No	246	376	1	1
**Using health workers (as source of information about malaria)**				
Yes	298	211	2.07 (1.56–2.68)	0.84 (0.60–1.20)
No	179	262	1	1
**Using friends (as source of information about malaria)**				
Yes	200	70	4.16 (3.04–5.68)	2.68 (1.79–4.01)*
No	277	403	1	1
**Using schools (as source of information about malaria)**				
Yes	99	46	2.43 (1.67–3.54)	1.30 (0.80–2.01)
No	378	427	1	1

* denotes an association where *p* < 0.05

### Factors associated with good practice to prevent malaria

The overall good practice for malaria prevention was 27.2% with 95%CI (24.4%.3–29.9%). The odds of having good practice increased by 1.93 times among participants who lived less than 2 months in the farming area as compared with participants who lived more than 2 months. Study participants who had media exposure as source of information had 1.64 times good practice in malaria prevention activities than their counter parts. (Table 4).

**Table 4:** Factors associated with good practice to prevent malaria among seasonal migrant farmworkers in Metema and West Armacheho districts, Northwest Ethiopia, 2018

Variables	Practice		COR with 95%CI	AOR with 95%CI
	Good	Poor		
**Age**				
13–20	46	198	0.53 (0.37–0.78)	0.51 (0.33–0.80)*
21–29	147	338	1	1
30–39	46	105	1.00 (0.97-o.68)	0.74 (0.45–1.19)
40–67	19	51	0.86 (0.49–1.50)	1.19 (0.61–2.35)
**Level of education**				
Unable to read and write	80	294	0.55 (0.31-o.82)	0.74 (0.39–1.40)
Able to read and write	22	51	0.80 (0.42–1.53)	0.7 (0.31–1.67)
Primary	122	284	0.80 (0.50–1.27)	1.17 (0.66–2.81)
Secondary and above	34	63	1	1
**Length of time living here in the current visit**				
< 2 months	178	398	1.64 (1.21–2.22)	1.93 (1.35–2.77)*
> 2 months	80	294	1	1
**Using mass media as source of health information**				
Yes	153	175	4.30 (3.18–5.82)	1.64 (1.30–2.60)*
No	105	517	1	1
**Using health workers as source of health information**				
Yes	186	323	2.95 (2.16–4.02)	1.06 (0.67–1.68)
No	72	369	1	1
**Using friends as source of health information**				
Yes	142	128	5.39 (3.95–7.36)	2.55 (1.61–4.02)*
No	116	564	1	1
**Using schools as source of health information**				
Yes	61	84	2.24 (1.55–3.23)	0.74 (0.44–1.36)
No	197	608	1	1
**Knowledge**				
Good	214	263	7.93 (5.54–11.36)	6.62 (4.46–9.83)*
Poor	44	429	1	1
**Attitude**				
Favorable	68	92	2.33 (1.64–3.32)	2.17 (1.40–3.37)*
Un favorable	190	600	1	1

## Discussion

The main aim of this study was to determine the knowledge and practice of malaria prevention and associated factors among migrant and seasonal farm workers**.** The results of this study showed that half and almost one fourth of the participants had good knowledge and practice respectively**.** Age, level of education, the length of stay in the farming area and mass media as a source of information about malaria were significantly associated with the knowledge and practice of malaria prevention.

This study showed that, 59.6% of the participants ever heard malaria related information from different sources. This finding was different from a study conducted in the same districts before 5 years, which showed 94.7% participants reported that they have heard of malaria [12]. This shows that there were different migrant and seasonal farm workers who visit the farming area from year to year. This finding is also by far different from a study conducted in Shewa Robit and Arbaminch zuria districts (Ethiopia) among general population, which showed that almost all the study respondents had ever heard of malaria [13, 14]. This finding was also lower than a study conducted in Western Cambodia [15]. This indicates that there is huge gap on malaria related information among migrant and seasonal farm workers in Ethiopia, which should be provided continuously at the departure and farming sites these population.

The present study revealed that the majority of the study participants were aware of major signs and symptoms of malaria (fever and headache), and mosquito bite causes malaria. However, only half of the study participants had an overall good knowledge about malaria prevention and control methods. This finding is different from a study conducted in Guarage zone (Ethiopia) among households, which showed that 86% of the participants had good knowledge on malaria prevention methods [16]. It is also by far lower than a study conducted in Woreta (Ethiopia), which showed that 95.8% of the study participants had knowledge of malaria transmission and prevention methods [17]. This indicates that malaria education is very low for these groups of population. Migrant and seasonal farmworkers are vulnerable groups for different diseases; at disproportionate risk of disease compared to non-migrant groups due to differences in social, economic, environmental, and institutional factors [18].

This study also showed that educational status was associated with the knowledge of malaria prevention methods. Participants whose educational status was secondary school and beyond had more knowledge about malaria. This finding is consistent with other studies conducted in Tigray (Ethiopia) and Kenya [19, 20]. This shows the importance of using different health communication strategies to deliver health education about malaria for the targeted group based on their educational status.

Length of stay in the farming area is another variable which was significantly associated with the knowledge and practice of the participants. The reasons might be those migrant and seasonal farm workers who passed more months at the farming areas become familiar with malaria messages, and this results in ignorance and loose motivation to practice malaria prevention strategies.

Age is also significantly associated with both knowledge and practice of malaria prevention. This finding is consistent with other studies [21, 22]. This might be because of the fact that as age increase, the exposure for knowledge and practice of malaria prevention also increase, but it does not mean that always true and scientific. Therefore, even if we need to give priorities for adolescent ages of these population, all age group of these population should be addressed with malaria prevention education programs.

This study revealed that only 27.2% of the participants had good practice in the prevention of malaria. Moreover, ITN utilization was only 20.74%. In fact, this was greater than a study which was conducted in the same districts before 5 years that showed only 8.9% of the participants used ITNs [12]. Even if the finding shows improvement of ITNs utilization comparing to previous years but still it is low compared with other studies in Ethiopia among the general population [23, 24]. This finding is also consistent with similar study populations in Thailand, Pakistan and India [25–27]. The main possible reason for this significant difference is the nature of their working condition (they often work at night and sleep on the farmlands, in the area where they were harvesting, directly on the ground on the straw of the crop) [28]. The other possible reason might be low accessibility of ITNs, poor knowledge and negative attitude towards ITNs utilization.

WHO strongly recommends that every suspected malaria case must be tested and every confirmed case must be treated with anti-malarial drugs in malaria endemic areas [29]. But this study showed that only 22.8% of the participants had treatment seeking behavior. This is lower than a study conducted in Tigray (Ethiopia) among household level in the general population in which 32.1% of participants seek treatment within 24 h from the onset of the illness. However, this finding is similar to other study in Myanmar with similar study populations [30]. The possible reasons for this low treatment seeking behavior might be the remoteness of their worksites, inaccessibility of health facilities and anti-malarial drugs, unaffordable cost charges [30] and self-medication with antimalarial drugs [27]. The implication of this finding indicates that continuous and intensive behavior change communication campaigns should be planned and implemented for these high risk populations at the farming areas.

This study also revealed that knowledge and attitude is significantly associated with practice of malaria prevention. This finding was similar with other studies conducted in Ethiopia and Senegal among adult population at household level [31, 32]. The implication of this finding is that, in order to increase ITNs utilization and other malaria prevention practices, we need to first increase the knowledge of malaria prevention methods and avoid misconceptions and wrong beliefs regarding the causes and prevention measures of malaria. Evidences also support the need of well-designed behavioral change programs to convert people’s knowledge and attitudes into practice [33].

According to the results of this study, those study participants who had exposure for mass media had better knowledge and practice of malaria prevention. This finding is supported by a cross sectional study conducted in sub-Saharan African countries which showed participants who reported not receiving malaria related information from radio and poster/ billboards results in low knowledge and practice to prevent malaria [34]. John Hopkins communication center’s malaria SBCC program also strongly supports the use of mass media in promoting malaria prevention and treatment behaviors [35]. The implication of this finding indicates that the Government’s policy makers and stakeholders should design mass media intervention as an effective approach for these targeted populations at their departure site and mass transportation area.

The strength of this study was using large sample size which increase its generalizability to other similar study populations in Ethiopia while its limitation include lack of measuring the prevalence and the incidence rate of malaria due to lack of fund for laboratory equipment. The other limitation of this study was because of the nature of the cross sectional design, this study was unable to establish causality. Moreover, the study may be exposed for social desirability bias and recall bias because of the inclusion of different attitudinal and experience related questions.

## Conclusion

In conclusion, the practice of malaria prevention among migrant and seasonal farm workers was low. Age, level of education, access to mass media and the length of stay in the farming site were factors significantly associated with the knowledge and practice of malaria prevention. Migrant and seasonal farm workers should be one of the priorities in the malaria prevention strategy in the country, and it is better to design and implement tailored malaria intervention programs and strategies for these hard to reach population.

## Data Availability

Data that support the findings are available from the correspondence author on reasonable request.

## Abbreviations

AOR: Adjusted odds ratio
CI: Confidence interval
DALY: Disability adjusted life years
ITN: Insecticide treated nets
MSFWs: Migrant and seasonal farm workers
PSUs: Primary sample units
WHO: World Health Organization
